# Dectin-1 Regulates IL-10 Production via a MSK1/2 and CREB Dependent Pathway and Promotes the Induction of Regulatory Macrophage Markers

**DOI:** 10.1371/journal.pone.0060086

**Published:** 2013-03-22

**Authors:** Suzanne E. Elcombe, Shaista Naqvi, Mirjam W. M. Van Den Bosch, Kirsty F. MacKenzie, Francesca Cianfanelli, Gordon D. Brown, J. Simon C. Arthur

**Affiliations:** 1 MRC Protein Phosphorylation Unit, College of Life Sciences, Sir James Black Complex, The University of Dundee, Dundee, Scotland, United Kingdom; 2 Section of Infection and Immunity, Institute of Molecular Sciences, School of Medicine and Dentistry, University of Aberdeen, Aberdeen, United Kingdom; 3 Division of Cell Signaling and Immunology, College of Life Sciences, Sir James Black Complex, The University of Dundee, Dundee, Scotland, United Kingdom; Friedrich-Alexander-University Erlangen, Germany

## Abstract

In response to infection by fungal pathogens, the innate immune system recognises specific fungal pathogen associated molecular patterns (PAMPs) via pattern recognition receptors including the C-type lectin dectin-1 and members of the Toll Like Receptor (TLR) family. Stimulation of these receptors leads to the induction of both pro- and anti-inflammatory cytokines. The protein kinases MSK1 and 2 are known to be important in limiting inflammatory cytokine production by macrophages in response to the TLR4 agonist LPS. In this study we show that MSKs are also activated in macrophages by the fungal derived ligand zymosan, as well as the dectin-1 specific agonists curdlan and depleted zymosan, via the ERK1/2 and p38α MAPK pathways. Furthermore, we show that MSKs regulate dectin-1 induced IL-10 production, and that this regulation is dependent on the ability of MSKs to phosphorylate the transcription factor CREB. IL-10 secreted in response to zymosan was able to promote STAT3 phosphorylation via an autocrine feedback loop. Consistent with the decreased IL-10 secretion in MSK1/2 knockout macrophages, these cells also had decreased STAT3 tyrosine phosphorylation relative to wild type controls after stimulation with zymosan. We further show that the reduction in IL-10 production in the MSK1/2 macrophages results in increased secretion of IL-12p40 in response to zymosan relative to wild type controls. The production of high levels of IL-10 but low levels of IL-12 has previously been associated with an M2b or ‘regulatory’ macrophage phenotype, which was initially described in macrophages stimulated with a combination of immune complexes and LPS. We found that zymosan, via dectin-1 activation, also leads to the expression of SphK1 and LIGHT, markers of a regulatory like phenotype in mouse macrophages. The expression of these makers was further reinforced by the high level of IL-10 secreted in response to zymosan stimulation.

## Introduction

While fungal infections in healthy individuals are relatively benign, in immuno-compromised patients they are frequently severe and are a major cause of morbidity and mortality. This represents an escalating problem as the number of people in these at-risk groups is increasing due to improved treatments for immunosuppressive diseases such as Human Immunodeficiency Virus, increases in the numbers of organ transplant recipients, and the use of immunosuppressive therapies for diseases such as cancer and autoimmune disorders.

Recognition of fungal pathogens by the innate immune system provides an initial defence against infection. Innate immune cells detect pathogens via the interaction of specific pathogen associated molecular patterns (PAMPs) with germline encoded pattern recognition receptors (PRRs) [1], [2], [3], [4], [5], [6]. Several groups of PRRs have been described, including Toll-like receptors (TLRs), NOD-like proteins, CARD-like helicases and members of the C-type lectin family such as dectin-1. At the onset of fungal infection, the fungal cell wall is the first structure to be seen by innate immune cells. Several components of the fungal cell wall are recognised, including phospholipomannan that can stimulate TLR2 and/or 4 and β-glucans that are recognised by the C-type lectin dectin-1 [1], [7], [8]. A key role for dectin-1 in the response to some fungal infections was first demonstrated by the finding that dectin-1 knockout mice are more susceptible to infection with *Candida albicans* or *Aspergillus fumigatus* [9], [10]. The importance of dectin-1 in mice however appears to relate to the species and strain of the fungal pathogen, as a 2^nd^ dectin-1 knockout model demonstrated decreased resistance to *Pneumocystis carinii* but not *Candida albicans* [11]. The role of dectin-1 is not restricted to mice as a homozygous mutation in the dectin-1 gene in humans has been shown to result in recurrent mucocutaneous candidiasis [12] while a Y238X polymorphism in the dectin-1 gene is associated with an increased incidence of *Candida* colonization in hematopoietic stem cell transplant patients [13]. In addition a role for dectin-1 has recently been found in the gut; mice lacking dectin-1 are sensitized to chemically induced colitis that correlated to an increased prevalence of pathogenic, compared to non-pathogenic, commensal fungi in the gut. A polymorphism in the human dectin-1 gene has also been correlated with the development of ulcerative colitis [14].

Zymosan, an extract from the cell wall of *Saccharomyces cerevisiae*, has been much used as a mimic for fungal stimulation in the innate immune system. Zymosan stimulates macrophages and dendritic cells via both dectin-1 and TLR2 [8], [10], [15], [16], [17], [18]. TLR2 is able to activate signalling via the recruitment of the Myd88 adaptor protein, leading to IL-1R-associated kinase (IRAK) activation, Lys63 linked poly-ubiquitination of Traf6 and activation of the Tak1 complex [4], [5], [6], [19], [20], [21], [22]. Tak1 in turn leads to the activation of both the NFκB and MAPK signalling cascades. Dectin-1 is activated by complex β-glucan containing structures. While single β-glucan molecules can bind to the C-type lectin domain of dectin-1, this is insufficient to activate signalling by dectin-1. Instead complex high molecular weight β-glucans are needed to promote receptor clustering and formation of a ‘phagocytic synapse’ before signalling pathways are activated [23]. Like TLRs, dectin-1 can activate both NFκB and MAPK cascades, however it signals through an ITAM-like motif in its cytoplasmic domain that recruits the tyrosine kinase Syk to activate downstream signalling [2], [24], [25].

While zymosan can induce the secretion of pro-inflammatory cytokines, it also strongly promotes the production of IL-10, a key anti-inflammatory cytokine that plays critical roles in controlling inflammation. Following infection, loss of IL-10 can give rise to an increased inflammatory reaction in response to the pathogen. While this in some circumstances may aid pathogen clearance, it can also give rise to cytokine induced tissue damage [26], [27]. The importance of IL-10 has been further underscored by the finding that knockout of IL-10 in mice causes inflammatory bowel disease due to a deregulated immune response to the gut flora [26], [27]. In line with this, mutation of IL-10 or the IL-10 receptor in humans have been correlated to a severe early onset form of colitis [28], [29].

The regulation of IL-10 transcription is complex and the importance of different promoter elements varies depending on the cell type and stimuli [26]. In macrophages roles for several transcription factors have been proposed in the regulation IL-10 transcription downstream of TLRs, including Sp1, CREB, NFκB [30], [31], [32], [33]. In addition, IL-10 induction can be sustained by an IFNβ feedback loop in macrophages [34].

Besides its role in TLR induced IL-10 production, CREB has also been proposed to regulate IL-10 transcription downstream of zymosan in macrophages and dendritic cells [35], [36]. The classical mechanism for CREB activation involves its phosphorylation on Ser133, however the kinase responsible for this phosphorylation is dependent upon the signal. For instance PKA phosphorylates CREB downstream of cAMP while a CaMK family member is thought to phosphorylate CREB downstream of Ca^2+^ signalling [37]. Alternatively, downstream of the ERK1/2 and p38α MAPK cascades, MSK1 and 2 are the major CREB kinases [38]. Zymosan activates both MAPK and Ca^2+^ signalling, and may also activate PKA via a prostaglandin mediated feedback loop [35], [36]. Thus the identity of the kinase required for CREB phosphorylation by zymosan is not clear.

MSKs are nuclear protein kinases that can be activated in cells downstream of either the ERK1/2 or p38α MAPK cascades [39], [40]. In addition to CREB, several substrates for MSKs have been identified, including the CREB-related transcription factor ATF1, as well as histone H3 and the p65RelA subunit of NFκB [38], [41], [42], [43]. Work with the TLR4 agonist LPS in macrophages has demonstrated an important role for MSKs in the transcription of IL-10 downstream of TLR signalling [32], [44]. Knockout of both MSK1 and 2 reduces IL-10 secretion by macrophages in response to LPS and as a result, MSK1/2 knockout macrophages produce elevated levels of IL-12 compared to wild type cells. Additionally MSKs also regulate the induction of another anti-inflammatory cytokine, IL-1ra, following LPS stimulation [45].

In this study we establish that MSK1 and 2 are activated downstream of dectin-1 and play a major role in dectin-1 mediated IL-10 production. In addition, we find that dectin-1 can stimulate the transcription of markers of regulatory macrophages in part via an IL-10 dependent mechanism.

## Methods

### Cell culture

Bone marrow derived macrophages (BMDMs) were prepared from mice as described [46]. Cells were maintained on bacterial grade plates for 7 days in DMEM supplemented with 10% heat inactivated FBS (Biosera), 2 mM L-glutamine, 100 units/ml penicillin G, 100 mg/ml streptomycin, 0.25 mg/ml amphotericin (Invitrogen) and 5 ng/ml M-CSF (R&D systems). Adherent cells were then re-plated on tissue culture grade plates in fresh media and used 24 hours after re-plating. Bone marrow derived dendritic cells (BMDCs) prepared from the bone marrow by differentiation for 7 days in DMEM supplemented with 10% heat inactivated FBS (Biosera), 2 mM L-glutamine, 100 units/ml penicillin G, 100 mg/ml streptomycin 0.25 mg/ml amphotericin (Invitrogen) and 5 ng/ml GM-CSF (R&D systems). Where indicated, cells were treated with 10 µM SB-747651A (kindly supplied by GlaxoSmithKline Pharmaceuticals R&D), 2 µM PD184352, 5 µM SB203580 or 4 µM Syk Inhibitor II (Calbiochem) for 1 hour before stimulation. Selectivity data for PD 184325, SB203580 and SB-747651A has been published previously [47], [48] while data for Syk Inhibitor II is shown in Fig S1. Cells were stimulated with either 200 µg/ml zymosan (E. coli O26:B6, Sigma L2654), 200 µg/ml depleted zymosan (Invivogen), 10 µg/ml curdlan (Sigma) or 100 ng/ml LPS (Sigma) for the indicated times. Cells were then lysed for either RNA extraction or immunoblotting.

Gene targeted mice with deletions in IL-10, MSK1, MSK2 and dectin-1 as well as a Ser133Ala knockin mutation in CREB have been described previously [10], [38], [41], [49], [50]. IL-10 and MSK1/2 knockout mice had been backcrossed to C57/Bl6 mice for at least 12 generations and CREB knockin mice for 6 generations. Dectin-1 knockouts were on either a C57/Bl6 or 129/SvJ background as indicated in the figure legends. Mice were maintained in accordance with UK and EU regulations, and work was covered by an appropriate home office license (60/3923) which was subject to review by the University of Dundee Ethical Review Committee.

### Quantitative RT-PCR

RNA was isolated using the RNeasy Microkit (Qiagen). Total RNA was reverse transcribed using iScript (BioRad), and real time PCR carried out using Sybrgreen based detection methods. Primers for the mRNA studies have been described previously [34]. For each PCR primer set the PCR product from the amplification of reverse transcribed total mRNA was cloned and its identity confirmed by DNA sequencing. For expression analysis 18S was used as an internal control and fold induction was determined from Ct values using the equation: 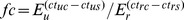
where fc is the fold change, E is the efficiency of the PCR, ct is the threshold cycle, u is the mRNA of interest, r is the reference gene (18s RNA), s is the sample and c is the un-stimulated control sample.

### Immunoblotting

BMDMs were lysed directly into SDS sample buffer and aliquots run on 10% polyacrylamide gels using standard methods. Proteins were transferred onto nitrocellulose membranes and specific proteins detected by immunoblotting. Antibodies against phospho-ERK1/2, phospho-p38, phospho-T581 MSK1, phospho-Y705 STAT3, total ERK1/2, total p38α, total STAT3 and GAPDH were from Cell Signaling Technology. The monoclonal antibody against phospho-Ser133 CREB was from Millipore, while the antibody against total MSK1 has been described previously [38].

### Cytokine measurements

The amount of cytokines secreted into the cell culture media was determined using a Luminex based technology using multiplex reagents from Bio-Rad according to the manufacturers protocols.

## Results

### Zymosan induces IL-10 secretion and MAPK activation in BMDMs

**Figure 1:**
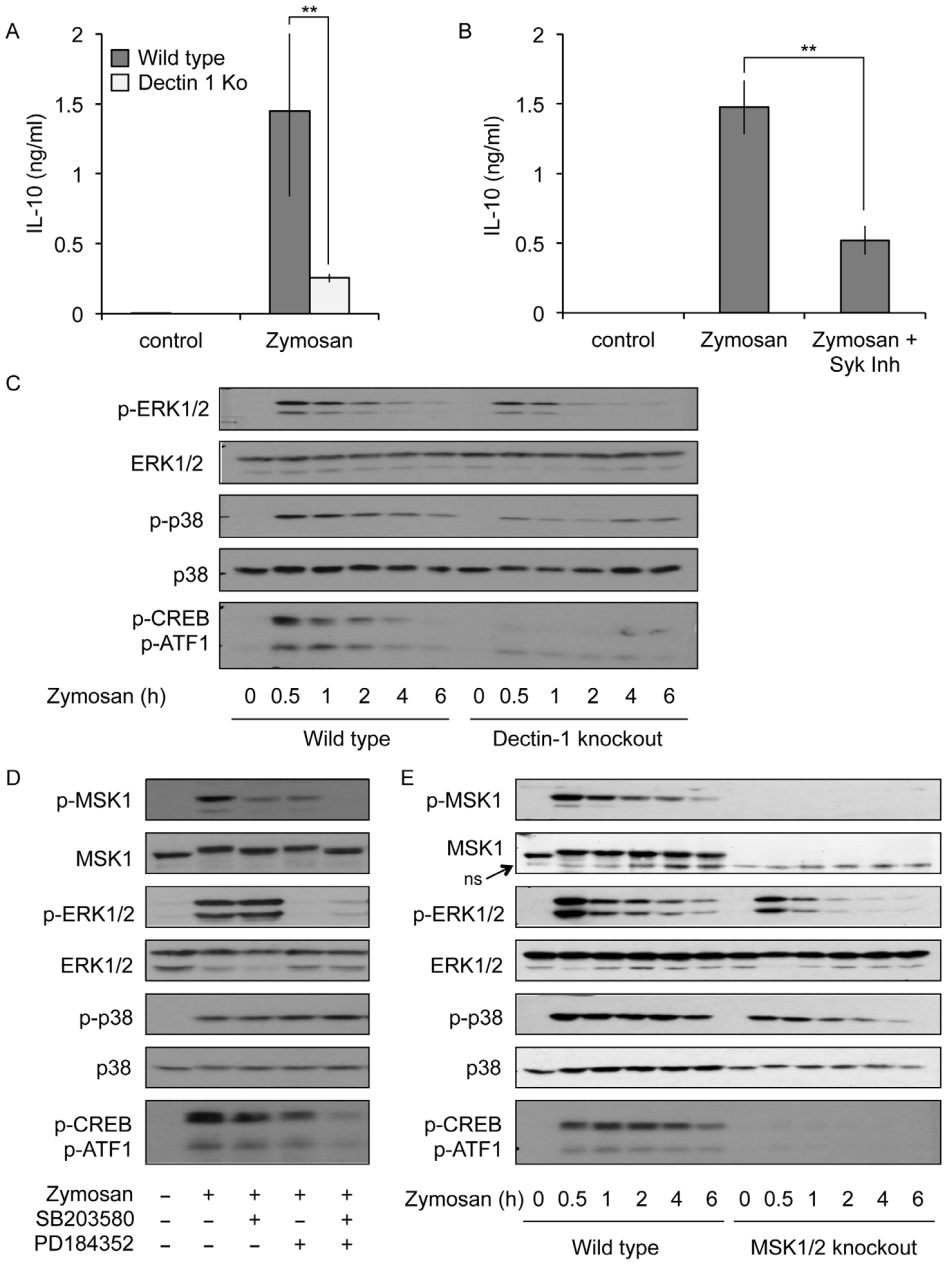
Zymosan induced MAPK activation and IL-10 production in part through dectin-1. (A) BMDMs were isolated from wild type or dectin-1 knockout animals on a C57/Bl6 background and stimulated with 200 µg/ml zymosan for 8 h. The levels of IL-10 protein secreted into the media were measured. (B) Wild type BMDMs were treated with 4 µM Syk Inhibitor II where indicated for 1 h before stimulation with 200 µg/ml zymosan for 8 h. Secreted IL-10 levels were then determined. In A and B error bars represent the standard deviation of independent cultures from 4 mice per genotype. P values (Student's t-test) of less that 0.001 are indicated by **. (C) BMDMs from wild type or dectin-1 knockout on a 129SvJ background were stimulated for the indicated times with 200 µg/ml zymosan. The levels of phospho and total ERK1/2, phospho and total p38 and phospho CREB/ATF1 were then determined by immunoblotting. (D) Wild type BMDMs were treated with 2 µM PD184352 or 5 µM SB203580 where indicated for 1 h before stimulation with 200 µg/ml zymosan for 30 min. The levels of total and phospho (T581) MSK1, phospho and total ERK1/2, phospho and total p38α and phospho CREB/ATF1 were then determined by immunoblotting. (E) Wild type or MSK1/2 double knockout BMDMs were stimulated with 200 µg/ml zymosan for the indicated times, and protein levels determined as in (D). In addition to MSK1, the total MSK1 antibody also picks up a non-specific band (ns) that runs at a molecular weight just below MSK1. The blots shown are representative of one (C) or three (D and E) separate experiments.

The yeast cell wall extract zymosan is known to stimulate macrophages via a combination of TLRs and the C-type lectin dectin-1 [15]. Stimulation of BMDMs with zymosan strongly stimulates secretion of IL-10 in part via stimulation of dectin-1 [10], [16]. In agreement with this we found the secretion of IL-10 was significantly reduced by knockout of dectin-1 in BMDMs (Fig 1A). Dectin-1 couples to intracellular signaling pathways via the tyrosine kinase Syk [16]. Consistent with the dectin-1 knockout results, in wild type cells zymosan induced IL-10 secretion was reduced by inhibition of Syk (Fig 1B).

### Dectin-1 stimulates MSK1 activation via both ERK1/2 and p38α MAPK

Downstream of TLR4, IL-10 induction has been shown to be regulated by the kinases MSK1 and 2, which phosphorylate the transcription factor CREB on the IL-10 promoter [32]. We therefore examined the possibility that dectin-1 could regulate MSK activation. MSKs are activated following phosphorylation by either ERK1/2 or p38α. Zymosan was able to activate both the ERK1/2 and p38α MAPKs, as judged by dual phosphorylation of the Thr-Xaa-Tyr motifs that correlates with the activation of these kinases (Fig 1C). In response to zymosan, the phosphorylation of p38α, and to a lesser extent ERK1/2, was reduced but not abolished by knockout of dectin-1. The MSK1/2 substrate CREB also became phosphorylated in response to zymosan and this was reduced by the knockout of dectin-1. ATF1 is a closely related transcription factor to CREB. The Ser133 phosphorylation site in CREB is conserved in ATF1 and is also phosphorylated by MSKs. ATF1 phosphorylation is detected by the phospho-CREB antibody, and in response to zymosan its phosphorylation mirrored that of CREB (Fig 1C). As zymosan activates both ERK1/2 and p38α, we used small molecule inhibitors of these two pathways to determine if one or both of these cascades were required to activate MSK1 and phosphorylate CREB. As a readout for MSK activation we analyzed the phosphorylation of the MSK1 on Thr581. In cells, Thr581 can be directly phosphorylated by ERK1/2 and/or p38α depending on the stimuli and this is required for MSK1 activation [39], [40]. ERK1/2 are activated in cells by MKK1/2. PD184352, a selective inhibitor of MKK1/2 [47], blocked the phosphorylation and activation of ERK1/2 in cells (Fig 1D). PD184352 also reduced, but did not block, MSK1 and CREB phosphorylation. The p38α/β inhibitor SB203580 had a similar effect to PD184352. A combination of both PD184352 and SB203580 repressed MSK1 and CREB phosphorylation more strongly than either inhibitor alone, indicating that both ERK1/2 and p38α contribute to MSK activation downstream of zymosan (Fig 1D). While CREB is an *in vivo* substrate for MSKs, it can also be phosphorylated by other kinases, such as PKA, on the same site [37]. To confirm that zymosan induced CREB phosphorylation via MSKs, BMDMs from MSK1/2 knockout mice were analyzed. Double knockout of MSK1 and 2 did not prevent ERK1/2 and p38α activation but did abolish CREB and ATF1 phosphorylation (Fig 1E).

**Figure 2:**
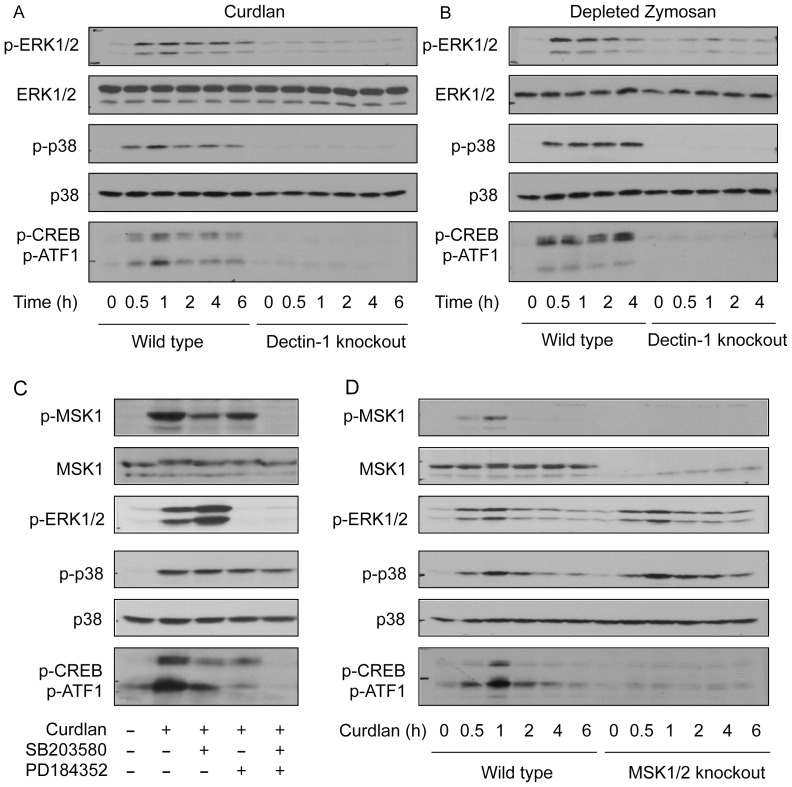
Dectin-1 specific ligands activate MSKs. (A) BMDMs from wild type or dectin-1 knockout on a 129SvJ background were treated with 10 µg/ml of curdlan for the indicated times and the levels of phospho and total ERK1/2, phospho and total p38α and phospho CREB/ATF1 determined by immunoblotting. (B) As (A) except cells were stimulated with 200 µg/ml of depleted zymosan. (C) Where indicated, wild type BMDMs were treated with 2 µM PD184352 or 5 µM SB203580 for 1 h. Cells were then stimulated with 10 µg/ml of curdlan for 30 min and the levels of total and phospho (T581) MSK1, phospho ERK1/2, phospho and total p38α and phospho CREB/ATF1 determined by immunoblotting. (D) Wild type or MSK1/2 knockout BMDMs were stimulated with 10 µg/ml of curdlan for the indicated times and the levels of the indicated proteins determined by immunoblotting. The blots shown are representative of one (B) or two (A, C, D) separate experiments.

Zymosan induced MAPK activation is not completely abolished by dectin-1 knockout (Fig 1C), due to the ability of zymosan to stimulate TLRs. Therefore we also examined CREB phosphorylation in response to ligands reported to be dectin-1 specific [15], [51]. Curdlan consists of purified β-glucan from *Alcaligenes faecalis*. Stimulation of BMDMs with 10 µg/ml curdlan stimulated both ERK1/2 and p38α phosphorylation, and this was abolished by knockout of dectin-1 (Fig 2A). Depleted zymosan, a preparation of zymosan treated with hot alkali to remove TLR2 agonists gave similar results. For stimulation with either curdlan or depleted zymosan, dectin-1 knockout greatly reduced the phosphorylation of the MSK substrates CREB and ATF1 (Fig 2A–B). Similar to what was observed for zymosan, in response to curdlan stimulation a combined inhibition of both ERK1/2 and p38α was required in order to block MSK1 phosphorylation on Thr581 as well as the phosphorylation of CREB and ATF1 (Fig 2C). Analysis of MSK1/2 knockout BMDMs again confirmed that CREB and ATF1 were phosphorylated by MSKs downstream of curdlan, and that MSK1/2 knockout did not block ERK1/2 or p38α activation (Fig 2D).

### ERK1/2 can have both positive and negative roles in regulating IL-10 mRNA transcription

**Figure 3:**
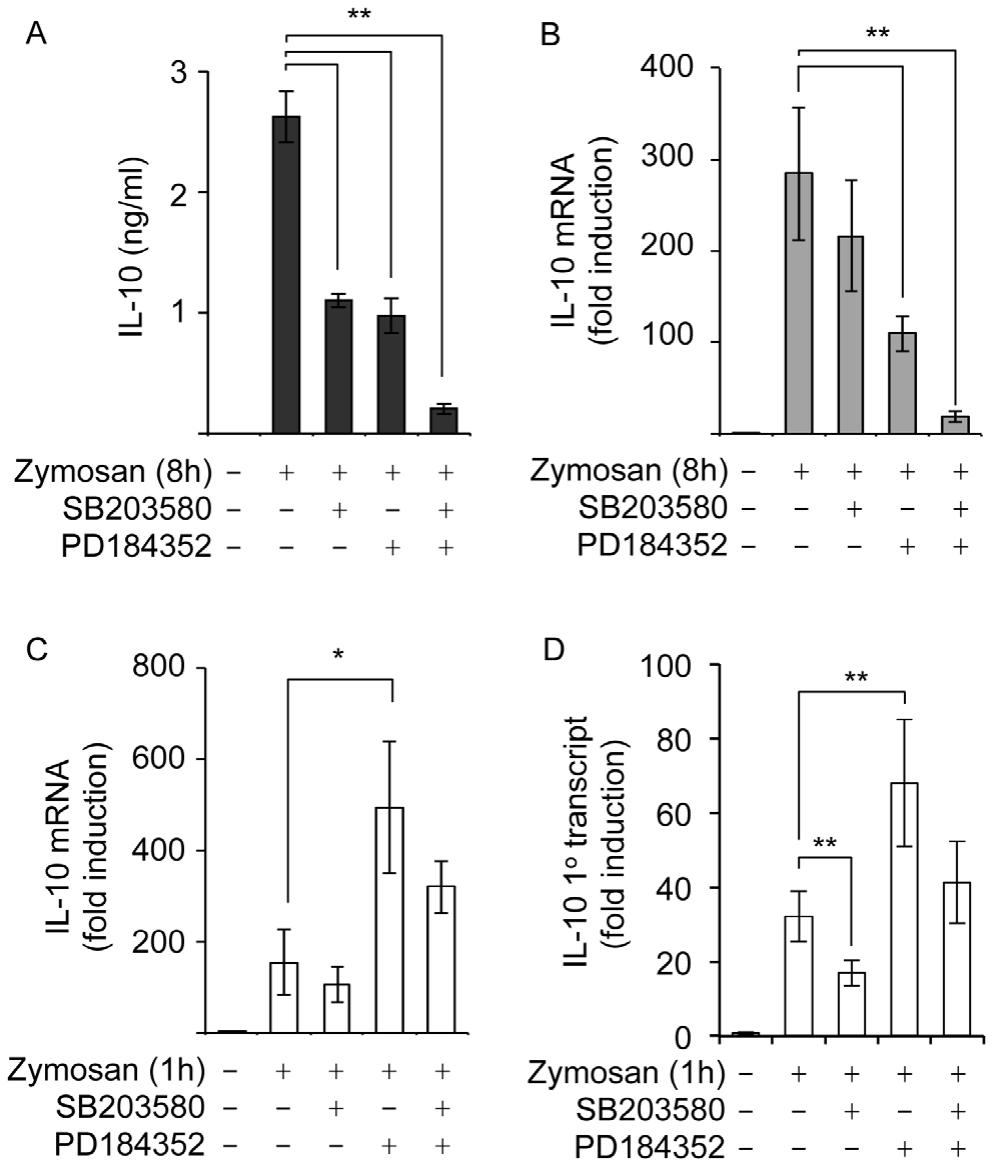
Inhibition of MAPK pathways modulate IL-10 induction in response to zymosan. Wild type BMDMs were treated with 2 µM PD184352 or 5 µM SB203580 as indicated. Cells were then stimulated for a further 1 or 8 h with 200 µg/ml of zymosan. IL-10 levels secreted into the media were measured at 8 h (A), while IL-10 mRNA levels were determined by qPCR at 1 (B) and 8 (C) h after stimulation. IL-10 primary transcript levels were determined by qPCR following 1 h of stimulation. Error bars represent the standard deviation of independent cultures from 4 mice per genotype. P values (Student's t-test) of less that 0.05 are indicated by * and less than 0.001 by **.

The above results indicate that dectin-1 contributes to IL-10 production in response to zymosan and that dectin-1 was able to activate the CREB kinase MSK1. To examine the role of MAPK signaling and MSKs in IL-10 induction we used a combination of small molecule inhibitors and mouse knockouts. The inhibitor PD184352 and SB203580 have been used extensively to study the cellular roles of the ERK1/2 and p38 MAPK cascades respectively. Multiple previous studies have used these compounds in BMDMs without reported issues with toxicity (for example [32], [45], [52]), and in line with this zymosan was still able to induce TNFα mRNA in the presence of these inhibitors (Fig S2). Several previous studies have suggested a role for ERK1/2 in zymosan induced IL-10 production [35], [53], [54]. Consistent with this we found that pre-treatment of BMDMs with PD184352 reduced IL-10 secretion following an 8 h treatment with zymosan. The p38α/β inhibitor SB203580 also inhibited zymosan induced IL-10 secretion, while a combination of both PD184352 and SB203580 resulted in a greater repression of IL-10 than either inhibitor alone (Fig 3A). Zymosan induced an increase in IL-10 mRNA levels and this increase was still sustained 8 h after stimulation (Fig 3B–C). When measured at 8 h, the effect of inhibiting MAPKs on IL-10 mRNA levels mirrored the effects seen on the levels of secreted IL-10 (Fig 3C). PD184352 treatment decreased the induction IL-10 mRNA by zymosan, while SB203580 showed a trend for a reduction in IL-10 mRNA although this did not reach statistical significance (p<0.05). A combination of both inhibitors however had an additive effect on IL-10 mRNA levels (Fig 3B). In contrast, following 1 h of stimulation with zymosan the effects of inhibiting MAPKs where different to that seen at 8 h (Fig 3C). At 1 h, blocking ERK1/2 activation with PD184352 actually increased rather than decreased IL-10 mRNA levels (Fig 3B). Inhibition of p38 on its own had little effect of IL-10 mRNA levels at this time. In combination with PD184352, SB320580 did however reduce IL-10 mRNA levels relative to PD184352 alone (Fig 3B). While the increase in IL-10 mRNA seen at 1 h suggests that IL-10 transcription is being activated, changes in mRNA levels could also result from a change in mRNA turnover. Therefore the induction of the primary transcript for IL-10 was also measured, as this provides a better indication of the changes in transcription. Zymosan stimulation at 1 h was able to stimulate the induction of the primary transcript for IL-10 and the pattern of inhibition by the MAPK inhibitors mirrored that seen for IL-10 mRNA (Fig 3D).

**Figure 4:**
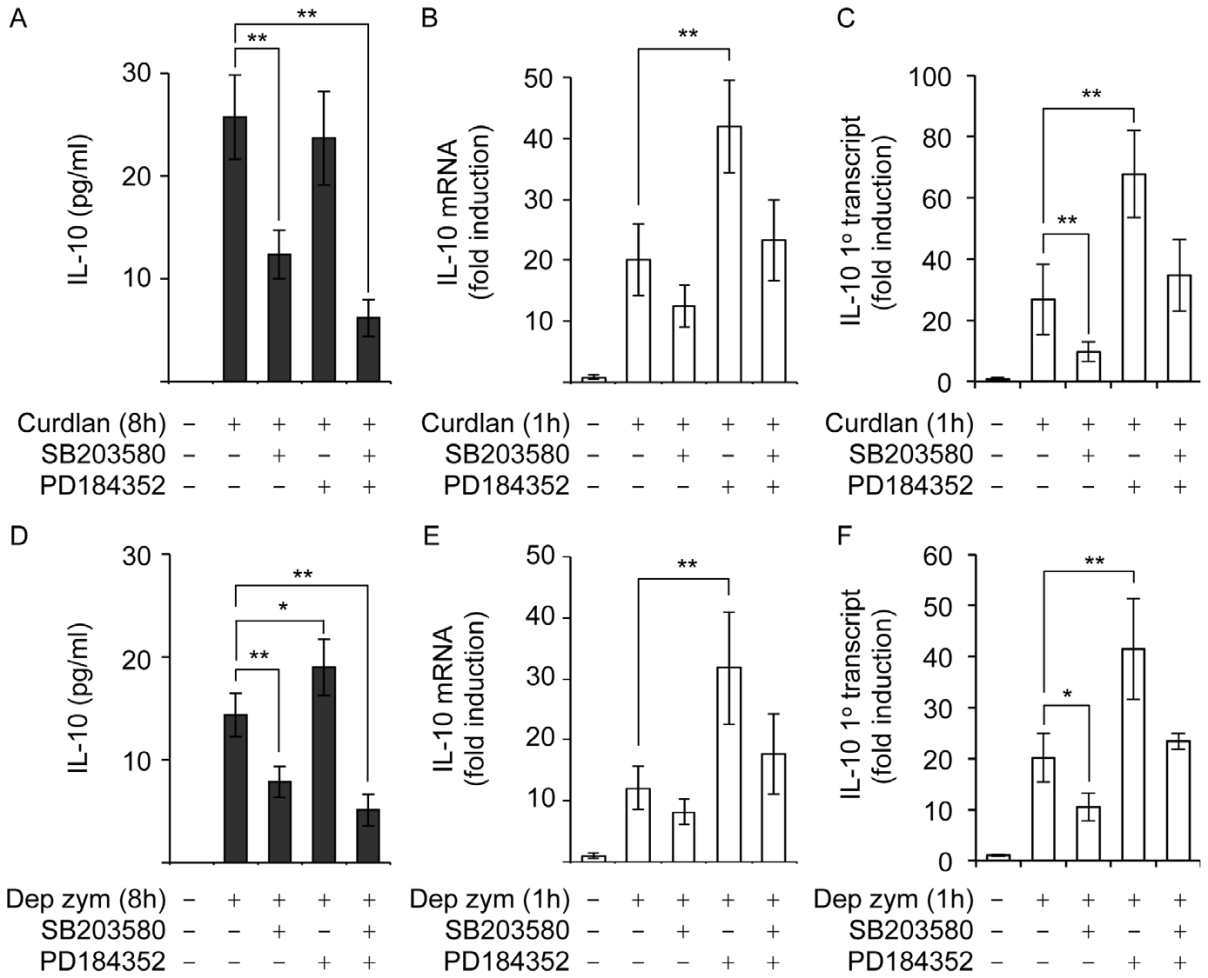
Inhibition of MAPK pathways modulates IL-10 induction in response to dectin-1 selective ligands. Wild type BMDMs were treated with 2 µM PD184352 or 5 µM SB203580 as indicated. Cells were the stimulated with 10 µg/ml curdlan. IL-10 secretion was measured after 8 h (A). Alternatively at 1 h IL-10 mRNA (B) or primary transcript (C) levels were determined by qPCR. (D–F) As above, except cells were stimulated with 200 µg/ml depleted zymosan. Error bars represent the standard deviation of independent cultures from 4 mice per genotype. P values (Student's t-test) of less than 0.05 are indicated by * and less than 0.01 by **.

To see if this effect of blocking ERK1/2 activation occurred with dectin-1 specific ligands, the induction of IL-10 in response to curdlan (Fig 4A–C) or depleted zymosan (Fig 4D–F) was examined. Both curdlan and depleted zymosan were able to induce IL-10 secretion, however the levels found in the media were much lower than seen for zymosan (Fig 4A, D). Similar to zymosan, a combination of both PD184352 and SB203580 was able to reduce IL-10 secretion in response to curdlan or depleted zymosan. Inhibition of p38 alone with SB203580 also reduced IL-10 secretion, however not to the same extent as a combination of both inhibitors. In contrast, PD184352 alone had little effect on curdlan induced IL-10 secretion and caused a small increase in IL-10 production in response to depleted zymosan (Fig 4A, D). The induction of IL-10 mRNA by depleted zymosan or curdlan was more transient than seen with zymosan, so the effects of MAPK inhibition were only examined at 1 h. Similar to what was seen with zymosan, inhibition of ERK1/2 activation with PD184352 increased the induction of IL-10 mRNA in response to a 1 h treatment with either curdlan or depleted zymosan (Fig 4B, E). As for zymosan, analysis of the primary transcript levels for IL-10 in response to curdlan or depleted zymosan gave a similar pattern to that seen for IL-10 mRNA (Fig 4C, F).

### MSKs promote IL-10 transcription downstream of dectin-1

**Figure 5:**
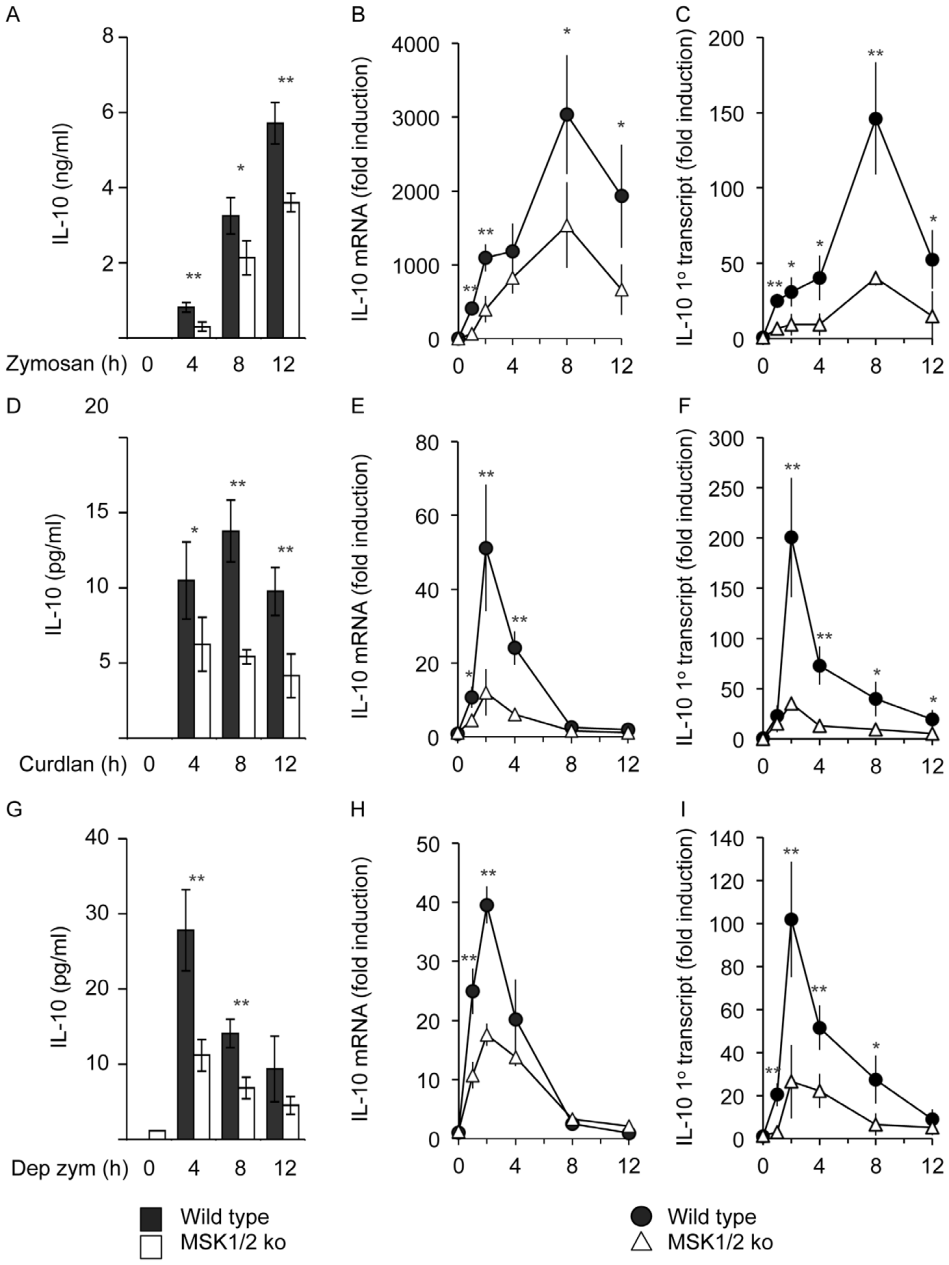
MSKs promote IL-10 transcription. Wild type and MSK1/2 knockout BMDMs were isolated and stimulated with either 200 µg/ml of zymosan (A, B, C), 10 µg/ml curdlan (D, E, F) or 200 µg/ml of depleted zymosan (Dep Zym, G, H, I) for the indicated times. IL-10 protein levels secreted into the media were measured (A, D, G) and IL-10 mRNA (B, E, H) or IL-10 1° transcript (C, F, I) induction determined by qPCR. Error bars represent the standard deviation of independent cultures from 4 mice per genotype. P values (Student's t-test) of less than 0.05 are indicated by * and less than 0.01 by **. ns indicates a p value of more than 0.05.

In response to TLR stimulation, maximal IL-10 production had been shown to be dependent on MSK activation. The above results could suggest that dectin-1 regulation of IL-10 may not require MSKs, as a combination of PD184352 and SB203580 blocks MSK activation (Fig 1 and 2) but not IL-10 transcription (Fig 3 and 4). They would however also be consistent with a model by which ERK1/2, in combination with p38α, exerted a positive effect on IL-10 transcription via MSKs but also inhibited IL-10 transcription via 2^nd^ mechanism independent of p38α and MSKs. To test the involvement of MSKs we used BMDMs isolated from MSK1/2 double knockout mice. In response to zymosan, MSK1/2 knockout macrophages secreted less IL-10 compared to wild type cells (Fig 5A). In line with this, the induction of IL-10 mRNA by zymosan was also lower in MSK1/2 knockout cells and decrease was maintained over a 12 h time course. Significantly the decrease in IL-10 mRNA observed MSK1/2 knockouts could be seen as early as 1 h, the time point at which PD184352 increased IL-10 mRNA levels (Fig 5B). Analysis of the induction of the primary transcript for IL-10 also showed a decrease in the MSK1/2 knockout cells relative to wild type BMDMs (Fig 5C).

Similar results were also obtained when the dectin-1 specific ligands curdlan and depleted zymosan were used (Fig 5D–I). MSK1/2 knockout resulted in decreased IL-10 secretion in response to curdlan and depleted zymosan (Fig 5D, G). The induction of IL-10 mRNA in response to curdlan or depleted zymosan was transient and was reduced by MSK1/2 knockout (Fig 5E, H). Importantly this reduction in IL-10 mRNA levels in the MSK1/2 knockout cells relative to wild type macrophages was apparent following 1 h of stimulation. Similar results were also obtained for the induction of the IL-10 primary transcript (Fig 5 F, G). MSK1/2 knockout did not however block the induction of all genes as for example TNFα mRNA induction in response to zymosan, curdlan or depleted zymosan was normal in MSK1/2 knockout BMDMs.

**Figure 6:**
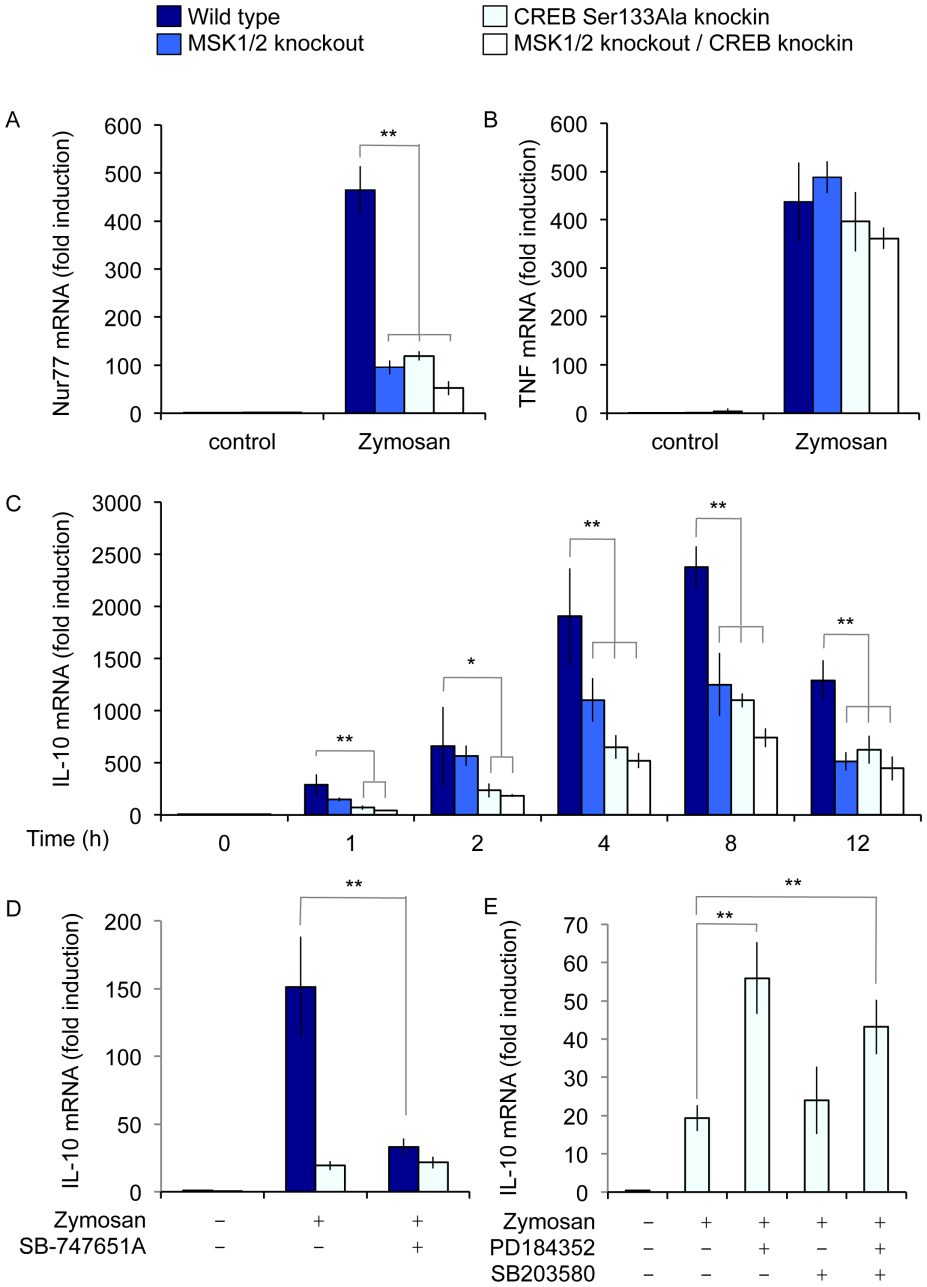
MSKs regulate IL-10 mRNA transcription via CREB. BMDMs were cultured from wild type, MSK1/2 knockout, CREB S133A knockin or MSK1/2 knockout/CREB S133A knockin mice. Cells were then stimulated with 200 µg/ml zymosan for either 1 h or for the indicated times. The induction of nur77 (A), TNFα (B) and IL-10 (C) mRNA was determined by qPCR. (D) Wild type or MSK1/2 knockout BMDMs were pretreated with 10 µM SB-747651A for 1 h where indicated and then stimulated with zymosan for 1 h. IL-10 mRNA induction was determined by qPCR. (E) MSK1/2 knockout BMDMs were treated with 2 µM PD184352 or 5 µM SB203580 as indicated before stimulation with zymosan for 1 h. IL-10 mRNA induction was then determined by qPCR. Error bars represent the standard deviation of independent cultures from 4 mice per genotype. In A to C, a P value (Student's t-test) relative to the wild type cells of less than 0.05 is indicated by * and less than 0.01 by **. In D and E a P value of less than 0.01 relative to the no inhibitor control is indicated by **.

MSKs phosphorylate the transcription factor CREB on Ser133 and are thus able to promote the transcription of CREB dependent genes [55]. In line with the ability of zymosan to induce CREB phosphorylation (Fig 1), zymosan also induced the transcription of the CREB dependent immediate early gene nur77 (Fig 6A). The transcription of nur77 was greatly decreased in cells isolated from mice with a Ser133 to Ala knockin mutation in the endogenous CREB gene. MSK1/2 knockout, which blocks CREB phosphorylation, reduced nur77 mRNA induction to a similar extent (Fig 6A). This was not due to a general impairment of transcription as the induction of the CREB independent gene TNFα was unaffected by either the CREB knockin or MSK1/2 knockout (Fig 6B). In agreement with the model that MSKs regulate IL-10 transcription via the phosphorylation of CREB, the induction of IL-10 mRNA by zymosan was lower in the CREB knockin cells relative to wild type macrophages (Fig 6C). Consistent with CREB acting downstream of MSKs, when crossed onto a MSK1/2 knockout background, the CREB knockin did not affect IL-10 mRNA levels relative to MSK1/2 knockout alone (Fig 6C). The MSK1/2 double knockout cells showed a slightly greater reduction in IL-10 mRNA levels relative to the single CREB mutation, suggesting that MSKs may regulate IL-10 via both a CREB dependent and independent mechanism (Fig 6C). This could reflect compensation from ATF1 in the CREB Ser133Ala knockin, or a role for another MSK substrate, such as histone H3, in IL-10 transcription.

To confirm that changes in IL-10 induction in the MSK1/2 knockout or CREB knockin BMDMs was not due to a developmental issue in the knockout macrophages, the effect of a MSK inhibitor SB-747651A [48] was also examined. In agreement with the knockout data, SB-747651A was able to inhibit IL-10 mRNA induction following stimulation of wild type cells with zymosan (Fig 6D) but did not affect that ability of zymosan to induce TNFα mRNA levels (Fig S2). In line with SB-747651A acting on MSKs, it did not affect the residual stimulation of IL-10 mRNA in MSK1/2 knockout cells (Fig 6D).

Together these results indicate that while MSKs contribute to IL-10 transcription downstream of dectin-1, the effect of blocking MSK activation on the initial stages of IL-10 transcription is different to blocking ERK1/2, an upstream activator of MSK1 and 2. To confirm that ERK1/2 could have an MSK independent effect on IL-10 transcription, the effect of blocking ERK1/2 activation in MSK1/2 double knockout cells was examined. As in wild type cells, in the MSK1/2 knockouts PD184352 was able to increase zymosan induced IL-10 mRNA levels following 1 h of stimulation (Fig 6E).

**Figure 7:**
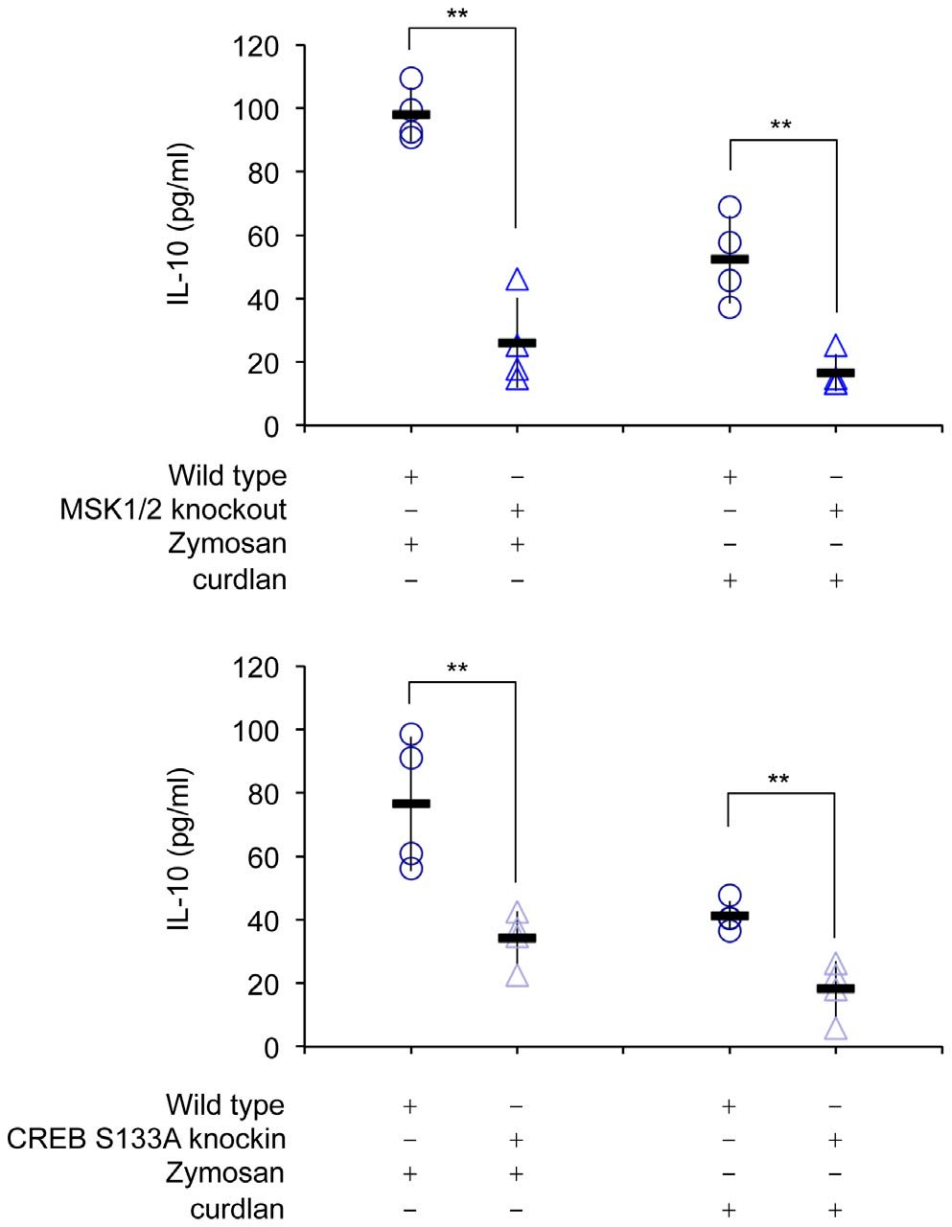
MSK and CREB regulate IL-10 secretion in BMDCs. (A) BMDCs were isolated from wild type and MSK1/2 knockout mice. Following stimulation with 200 µg/ml zymosan or 10 µg/ml curdlan for 8 h the levels of IL-10 secreted into the media was determined. (B) as (A) except BMDCs were from wild type or CREB Ser133Ala knockin macrophages. In both panels measurements were made for independent preparations of BMDCs from 4 mice per genotype. Individual measurements (open symbols) as well as average and standard deviation are shown. A p value (Student's t-test) of less that 0.01 is shown by **.

Other innate immune cells such as dendritic cells can also make IL-10 in response to stimulation with zymosan. In agreement with the effect of MSK1/2 knockout in BMDMs, MSK1/2 knockout also reduced the secretion of IL-10 in BMDCs following stimulation with either zymosan or curdlan (Fig 7A). A similar result was also seen with CREB Ser133Ala knockin macrophages (Fig 7B).

### MSK knockout increases IL-12p40 induction in response to zymosan

**Figure 8:**
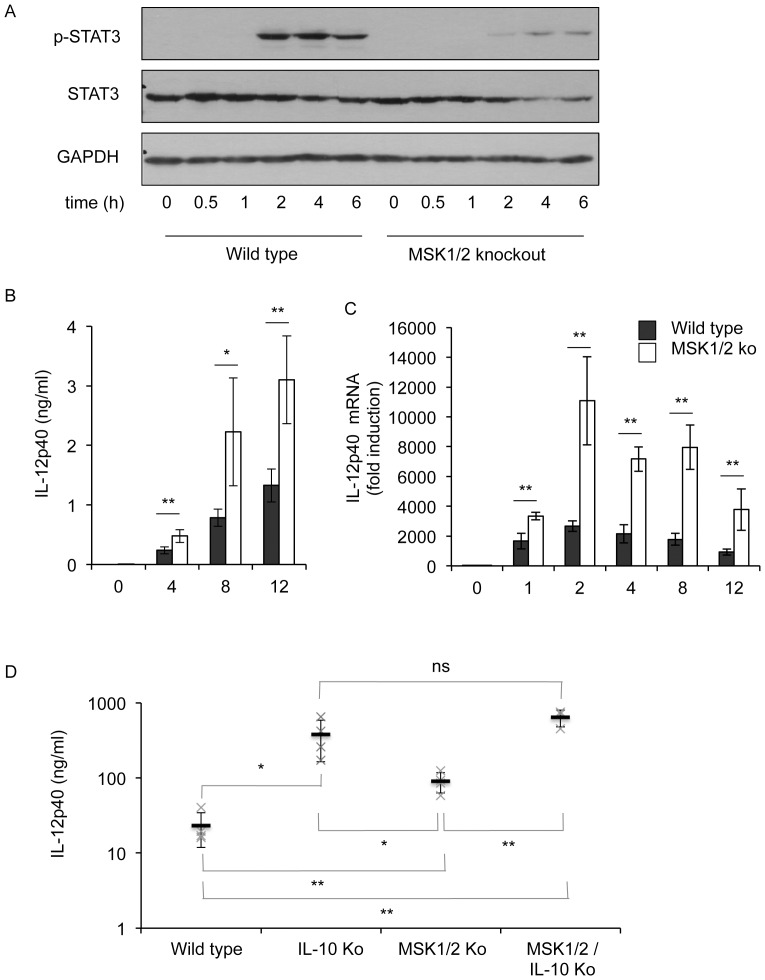
Zymosan induces STAT3 phosphorylation via IL-10. (A) BMDMs were isolated from wild type or MSK1/2 knockout mice and stimulated for the indicated times with 200 µg/ml zymosan. Cells were lysed and the levels of total and phospho (Y705) STAT3 as well as GAPDH were determined by immunoblotting. (B, C) BMDMs from either wild type or MSK1/2 knockout mice were stimulated for the indicated times with 200 µg/ml zymosan. Secreted IL-12p40 protein (B) and IL-12p40 mRNA (C) levels were determined. (D) Wild type, IL-10 knockout, MSK1/2 double knockout or MSK1/2/IL-10 triple knockout BMDMs were treated with 200 µg/ml zymosan for 8 h. Secreted IL-12p40 levels were then determined. The average for each genotype is shown by a bar and the BMDMs from individual mice by X. Error bars (B–D) represent the standard deviation of independent cultures from 4 mice per genotype. P values (Student's t-test) of less than 0.05 are indicated by * and less than 0.01 by **.

IL-10 is an anti-inflammatory cytokine and is able to repress the induction of IL-12 by macrophages via a STAT3 dependent mechanism [56]. Thus IL-10 secretion in response to PRR activation is able to set an autocrine feedback loop on macrophages that results in STAT3 phosphorylation. In line with the decreased IL-10 production by MSK1/2 knockout cells (Fig 5A), the phosphorylation of STAT3 on Tyr705 following zymosan stimulation was lower in MSK1/2 knockout BMDMs compared to wild type cells (Fig 8A). In response to zymosan BMDMs produced only very small amounts of IL-12p70 (data not shown), however they did produce measureable amounts of IL-12p40. The secretion of IL-12p40, as well as the induction of IL-12p40 mRNA, was increased in MSK1/2 knockout BMDMs relative to wild type cells following zymosan stimulation (Fig 8B, C). To confirm that IL-10 could repress the production of IL-12p40 following zymosan stimulation BMDMs were prepared from IL-10, MSK1/2 double knockout and MSK1/2/IL-10 triple knockout cells. Relative to wild type cells, IL-10 knockout BMDMs secreted more IL-12p40 in response to zymosan stimulation (Fig 8D). MSK1/2 double knockout also increased IL-12p40 secretion, although not to the same extent as IL-10 knockout. This may reflect the observation that the MSK1/2 knockout does not completely abrogate IL-10 secretion or STAT3 phosphorylation in response to zymosan stimulation (Fig 5, Fig 8A). No significant difference was seen in IL-12p40 levels between IL-10 single and MSK1/2/IL-10 triple knockout BMDMs, which would be consistent with MSKs affecting IL-12p40 secretion via an IL-10 dependent mechanism.

### Dectin-1 activation promotes regulatory markers in BMDMs

**Figure 9:**
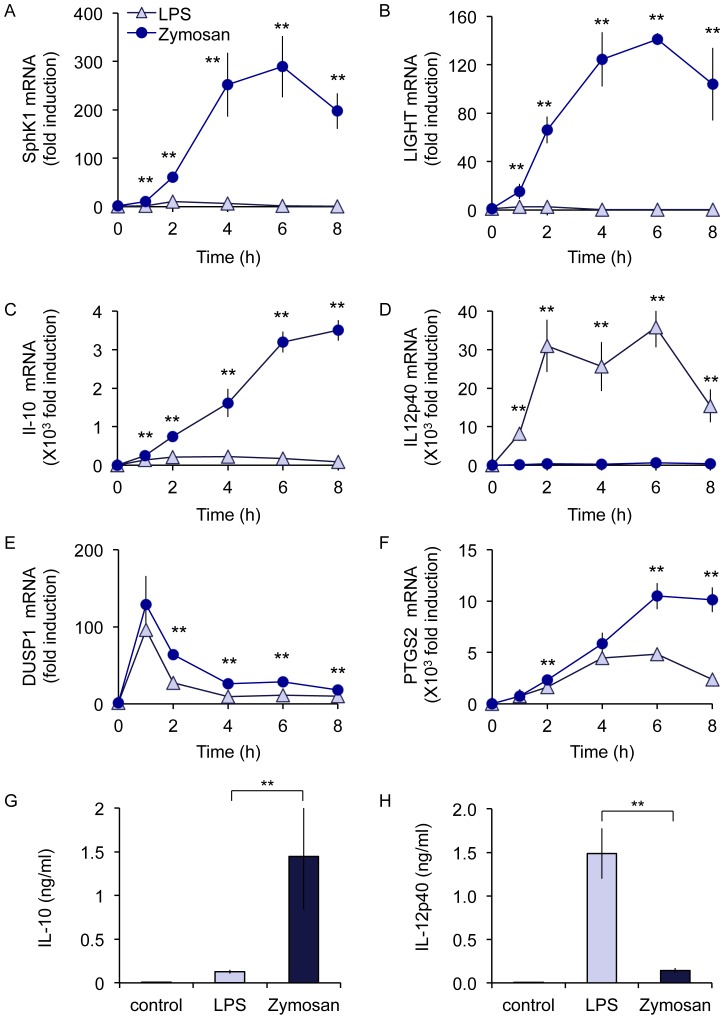
Zymosan induces the expression of regulatory macrophage markers. (A–H) Wild type BMDMs were stimulated with either 100 ng/ml LPS or 200 µg/ml zymosan for the indicated times. Total RNA was then isolated and the induction of SphK1 (A), LIGHT (B), IL-10 (C), IL-12p40 (D), DUSP1 (E) or PTGS2 (F) mRNA determined by qPCR. In addition the levels of IL-10 (G) and IL-12p40 (H) protein secreted into the media were measured at 8 h. Error bars represent the standard deviation of independent cultures from 4 mice.

The production of high levels of IL-10 but low levels of IL-12 have previously been shown to be a characteristic of regulatory or M2b macrophages [57], [58]. The induction of a regulatory like phenotype in macrophages has been associated with greatly increased expression of SphK1 and LIGHT mRNA [59]. LPS stimulation was found to be a poor stimulus for the transcription of either SphK1 or LIGHT. In contrast both SphK1 and LIGHT mRNA levels were greatly increased in response to zymosan (Fig 9A, B). This phenotype was also reflected in IL-10 mRNA levels; while at 1 h LPS and zymosan induced similar levels of IL-10 mRNA at later time points zymosan caused a higher and more sustained induction of IL-10 mRNA (Fig 9C). These differences were not reflected in all genes; for instance the induction of DUSP1 and PTGS2 mRNAs were more comparable between the two stimuli while LPS resulted in a much stronger induction of IL-12p40 mRNA (Fig 9D–G). In line with the mRNA data, LPS treatment resulted in more IL-12p40 secretion but less IL-10 secretion relative to zymosan (Fig 9G, H).

**Figure 10:**
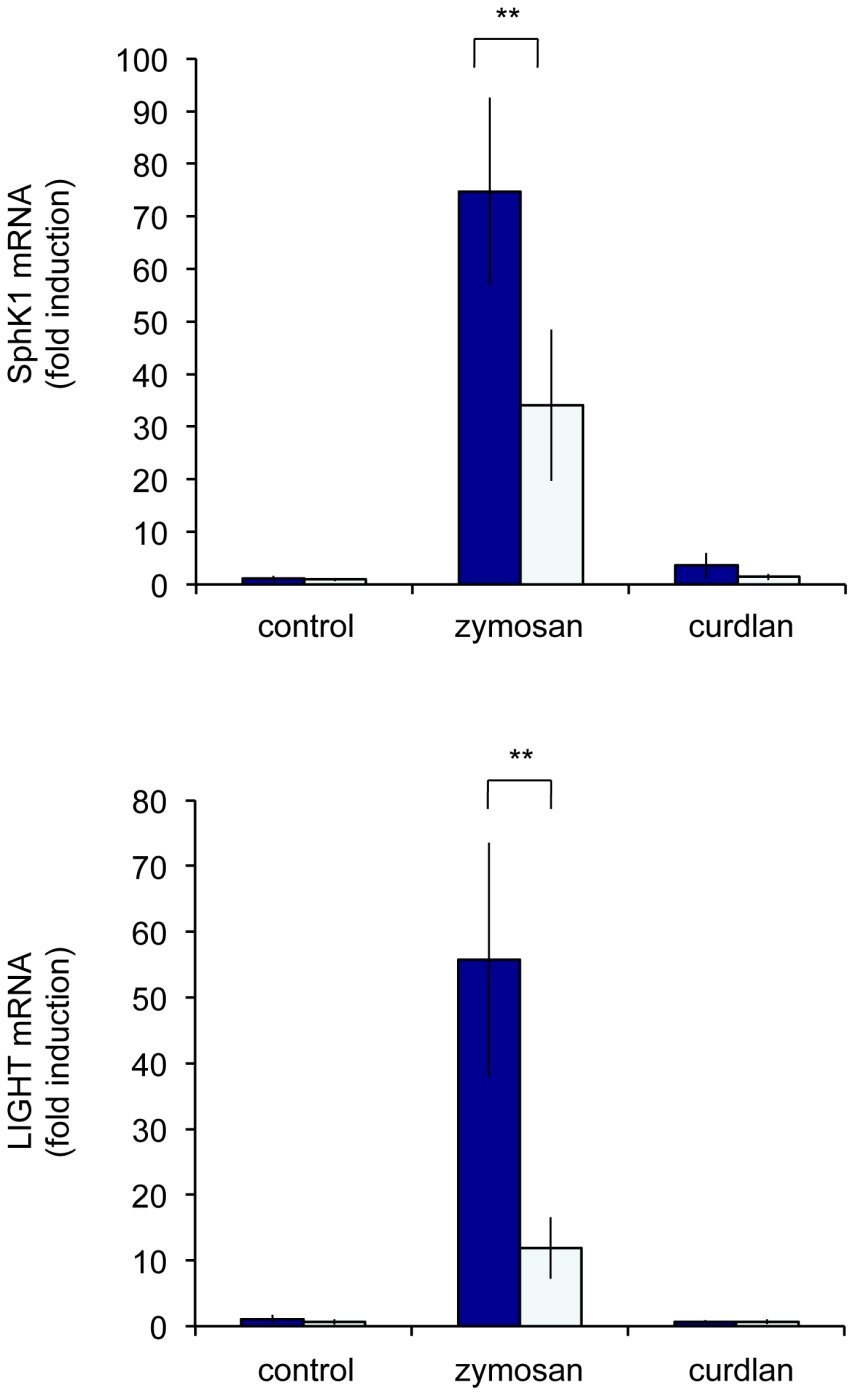
SphK1 and LIGHT mRNA induction is partially regulated via dectin-1. BMDMs were isolated from either wild type or dectin-1 mice on a C57/Bl6 background. Cells were stimulated with either 200 µg/ml zymosan or 10 µg/ml curdlan for 8 h. Total RNA was then isolated and SphK1 (A) and LIGHT (B) mRNA levels determined. Error bars represent the standard deviation of independent cultures from 4 mice per genotype. P values (Student's t-test) of less than 0.01 are indicated by **.

**Figure 11:**
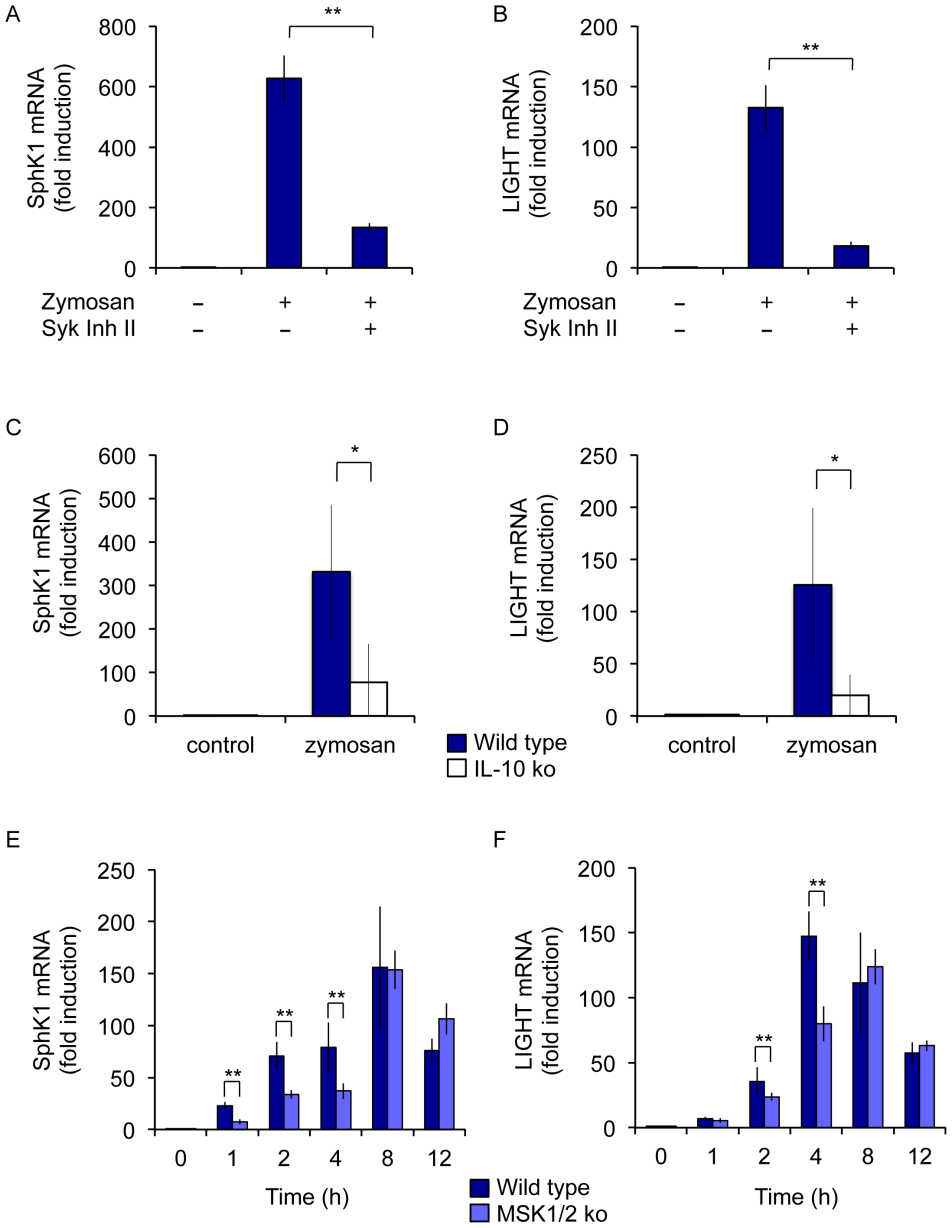
SphK1 and LIGHT mRNA induction is regulated by Syk and IL-10. (A, B) Wild type BMDMs were stimulated with 200 µg/ml zymosan in the presence or absence of 4 µM Syk Inh II. The induction of SphK1 (A) and LIGHT (B) mRNA was determined by qPCR. (C, D) Wild type or IL-10 knockout BMDMs were stimulated for 8 h with 200 µg/ml zymosan and the induction of SphK1 (C) and LIGHT (D) mRNA was determined by qPCR. (E, F) Wild type or MSK1/2 knockout BMDMs were stimulated for 8 h with 200 µg/ml zymosan and the induction of SphK1 (E) and LIGHT (F) mRNA was determined by qPCR. Error bars represent the standard deviation of independent cultures from 4 mice per genotype. P values (Student's t-test) of less than 0.05 are indicated by * and less than 0.01 by **.

The upregulation of SphK1 and LIGHT was further examined. The induction of both SphK1 and LIGHT was lower in dectin-1 knockout BMDMs relative to wild type cells (Fig 10), suggesting a role for dectin-1 in promoting the maximal induction of these genes. The inability of dectin-1 knockout to completely abolish the induction of these two genes suggests that other receptors activated by zymosan are involved in their regulation. In agreement with this the dectin-1 specific agonist curdlan was much less effective at promoting SphK1 and LIGHT mRNA induction (Fig 10). To further analyze the pathways required for the induction of these mRNAs, cells were pretreated with a Syk inhibitor before stimulation with zymosan. Both the induction of SphK1 and LIGHT mRNA was decreased by the Syk inhibitor (Fig 11A, B). The induction of these genes was also partially dependent on IL-10, as the induction of both SphK1 and LIGHT was reduced, but not abolished, by the knockout of IL-10 (Fig 11C, D). Finally we examined the effect of MSK1/2 knockout on SphK1 and LIGHT mRNA induction. At early time points following zymosan stimulation there was a partial reduction in both SphK1 and LIGHT mRNA induction in the MSK1/2 knockout BMDMs relative to wild type macrophages, however this was not maintained at later time points (Fig 11E, F).

## Discussion

We show here that MSKs and CREB are directly involved in regulating IL-10 transcription downstream of dectin-1. Stimulation of macrophages with either dectin-1 specific ligands or the combined dectin-1/TLR agonist zymosan stimulated CREB phosphorylation. As discussed above, several kinases are able to phosphorylate CREB on Ser133 in cells, however through the use of MSK1/2 double knockouts we demonstrate that MSKs are the major CREB kinases downstream of dectin-1. MSK1/2 knockout or MSK1/2 inhibitors as well as mutation of the Ser133 phosphorylation site in CREB confirmed the importance of these proteins in regulating IL-10 transcription downstream of dectin-1. This provides the first direct evidence for MSK activation and its role in transcriptional regulation downstream of dectin-1.

**Figure 12:**
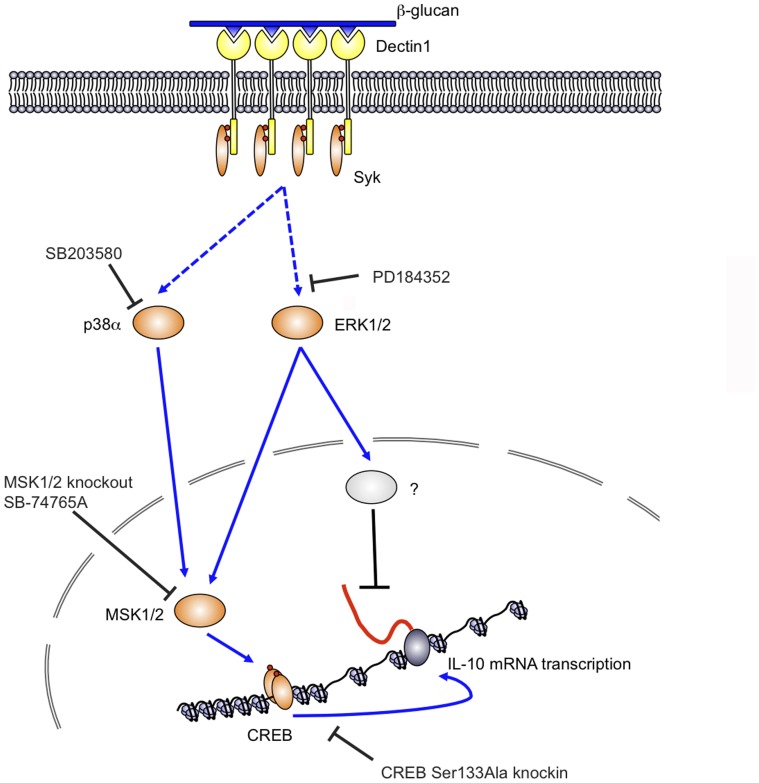
ERK1/2 regulate IL-10 transcription via MSK1/2 dependent and independent mechanisms. Dectin-1 is activated by complex β-glucan containing particles that induce clustering of dectin-1 at the membrane and formation of a phagocytic synapse. This leads to Syk recruitment to the ITAM like sequence in the cytoplasmic domain of dectin-1. Syk then mediates the activation of downstream signaling including the ERK1/2 and p38α MAPK cascades. Both ERK1/2 and p38α phosphorylate and activate the protein kinases MSK1 and 2. These in turn phosphorylate CREB on the IL-10 gene promoter, which stimulates IL-10 mRNA transcription. In addition, ERK1/2 also activate a MSK and p38 independent pathway that inhibits IL-10 mRNA transcription. The identity of this pathway is not clear. In addition to the ERK1/2 and p38α pathways shown dectin-1 also activates NFκB that likely also plays a role in inducing IL-10 transcription.

In response to dectin-1, we show that both ERK1/2 and p38α are able to phosphorylate and activate MSKs. The effects of small molecule inhibitors of the ERK1/2 or p38α pathways did not completely correspond to the effects of MSK1/2 knockout on IL-10. When IL-10 secretion or IL-10 mRNA levels were measured 8 h after zymosan addition, an inhibition could be seen in the presence of small molecule inhibitors of either the ERK1/2 or p38α pathways, while a combination of both inhibitors had an additive effect. This is in agreement with previous reports that have shown that inhibition of ERK1/2 activation reduces IL-10 secretion in macrophages and dendritic cells [35], [54]. Surprisingly a different picture was seen at early time points when IL-10 mRNA induction was measured (Fig 3 and 4). At 1 h, blocking ERK1/2 activation with the MEK1/2 inhibitor PD184352 promoted rather than inhibited IL-10 transcription. This was independent of MSKs as it still occurred in MSK1/2 knockout cells (Fig 6E). Thus at early time points ERK1/2 and p38 promote IL-10 transcription via MSKs while ERK1/2 also inhibits IL-10 transcription via a distinct mechanism (Fig 12). Further work will be required to determine the mechanism behind this ERK1/2 dependent inhibitory mechanism. Interestingly it is not the only example of when ERK1/2 inhibition increasing cytokine transcription. For example knockout of Tpl2, the MAP3K required for ERK1/2 activation by TLRs, resulted in elevated INFβ and IL-12 transcription in response to TLR4 and 9 agonists in macrophages and conventional dendritic cells [60]. A similar effect on IL-12 induction was reported in double knockouts of p38γ and δ, due to an unexpected requirement for these kinases to maintain Tpl2 protein levels in macrophages [61]. Significantly these effects were, at least in part, independent of IL-10 as the MKK1/2 inhibitor U0126 was still able to increase IFNβ and IL-12 production in IL-10 knockout cells [60]. The induction of c-fos by ERK1/2 was proposed as a mechanism to explain the inhibition of IFNβ and IL-12 production by ERK1/2 [60]. In line with this, transgenic overexpression of c-fos repressed TLR4 induced cytokine production in dendritic cells [62]. Upregulation of c-fos may however not explain the negative effects of ERK1/2 activity on IL-10 transcription as this paper demonstrated increased rather than decreased IL-10 secretion in the c-fos transgenic mice. However as these measurements were made at 6 or 24 hours after stimulation, a transient early repressive effect of c-fos on IL-10 transcription cannot be completely ruled out [62].

*In vivo*, macrophages have to play different roles depending on their situation. This has led to the concept that different stimuli or combinations of stimuli can polarize macrophages into distinct phenotypes [57], [58], [63], [64]. Relative to the TLR4 agonist LPS, zymosan induced a high level of IL-10 production but a low level of IL-12p40 (Fig 9). A similar cytokine profile has been linked to ‘regulatory’ or M2b polarized macrophages [59]. Regulatory macrophages, while retaining the ability to produce some pro-inflammatory cytokines have been suggested to limit inflammation and promote resolution; for example they are able to improve survival in mouse models of LPS induced endotoxic shock and EAE [65], [66], [67]. In mice, SphK1 and LIGHT have been proposed as markers for regulatory macrophages [59], and we found that stimulation via dectin-1 in response to zymosan was able to induce these genes (Fig 9). Regulatory macrophages were originally described following co-stimulation of macrophages with LPS and immune complexes, although other stimuli including apoptotic neutrophils and PGE2 have also been described to induce a similar phenotype in macrophages [58], [68], [69]. Interestingly both immune complexes, via Fcγ receptors, and dectin-1 can couple to downstream signaling via the tyrosine kinase Syk [70], [71]. Thus zymosan, which acts via a combination of TLRs and dectin-1 may activate similar signaling networks to those activated by a combination of immune complexes and LPS. The induction of both SphK1 and LIGHT was reinforced by an autocrine IL-10 feedback loop as demonstrated by the decreased induction of both these genes in IL-10 knockout BMDMs (Fig 11). The decreased, but not abolished, secretion of IL-10 in the MSK1/2 knockouts (Fig 5) may therefore explain the transient decrease in SphK1 and LIGHT mRNA induction in the MSK1/2 knockout macrophages (Fig 11).

In summary we report here that MSKs play important roles in regulating IL-10 production downstream of fungal PAMPS, suggesting that MSKs could play a role in the control of fungal infection. In addition, we provide evidence that in BMDMs dectin-1 may stimulate an ERK1/2 pathway that transiently antagonizes the action of IL-10.

## Supporting Information

Figure S1 **In vitro kinase selectivity of Syk Inhibitor II.** The effect of 0.1 or 1 mM Syk inhibitor II on the ability of a panel of kinases to phosphorylate their substrates was determined. Results are expressed as the % kinase activity remaining relative to a control reaction with no inhibitor. Data is shown as the average and variance of two measurements. The methods used in the kinase selectivity screen are described in Bain et al, Biochemical Journal, 2007, 408 (3); 297–315.
(PDF)

Figure S2 **Effect of MAPK inhibitors and MSK1/2 knockout on TNF mRNA Induction.** (A) Wild type BMDMs were treated with 2 µM PD184352 or 5 µM SB203580 as indicated. Cells were then stimulated for a further 1 h with 200 µg/ml zymosan. Total RNA was isolated and the levels of TNF mRNA determined by qPCR. (B) as (A) except cells were stimulated for 8 h with zymosan. (C) Wild type BMDMs were incubated for 1 h with 5 µM SB-747651A before stimulation with 200 µg/ml zymosan for a further 1 h. TNF mRNA levels were determined by qPCR. (D–F) Wild type or MSK1/2 knockout BMDMs were stimulated for between 1 and 12 h as indicated with either 200 µg/ml zymosan (D), 10 µg/ml curdlan (E) or 200 µg/ml depleted zymosan (F). Error bars represent the standard deviation of independent cultures from 4 mice per genotype.
(PDF)
