# Supplementary material for: Dectin-1 Regulates IL-10 Production via a MSK1/2 and CREB Dependent Pathway and Promotes the Induction of Regulatory Macrophage Markers
Source: PLoS One. 2013 Mar 22;8(3):e60086. doi: 10.1371/journal.pone.0060086 (PMC3606242; doi:10.1371/journal.pone.0060086)
Supplement: Figure S1 — In vitro kinase selectivity of Syk Inhibitor II. The effect of 0.1 or 1 mM Syk inhibitor II on the ability of a panel of kinases to phosphorylate their substrates was determined. Results are expressed as the % kinase activity remaining relative to a control reaction with no inhibitor. Data is shown as the average and variance of two measurements. The methods used in the kinase selectivity screen are described in Bain et al, Biochemical Journal, 2007, 408 (3); 297–315. (PDF) [file pone.0060086.s001.pdf]

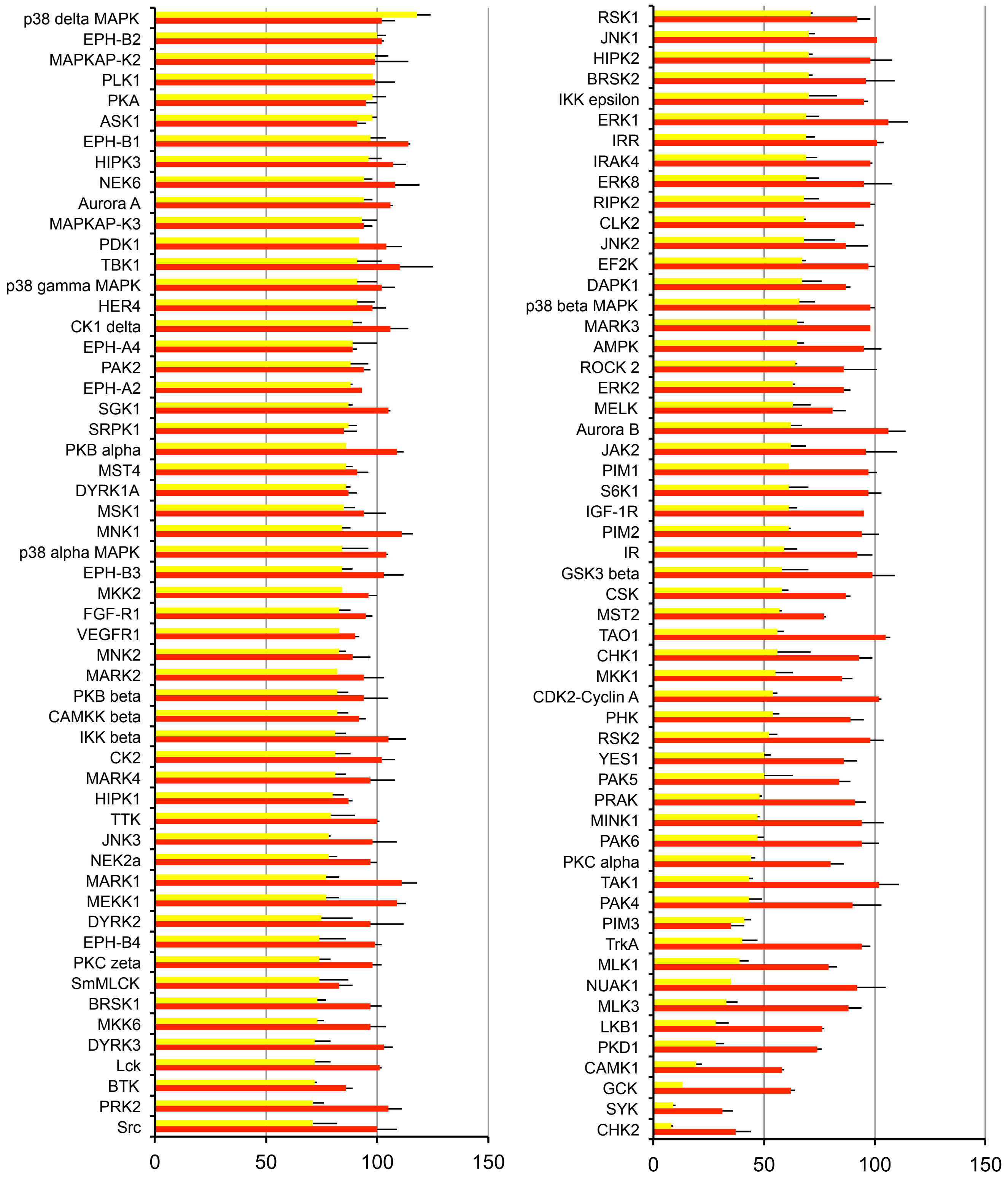

Figure S1. In vitro kinase selectivity of Syk Inhibitor II

The effect of 0.1 or 1 μM Syk inhibitor II on the ability of a panel of kinases to phosphorylate their substrates was determined. Results are expressed as the % kinase activity remaining relative to a control reaction with no inhibitor. Data is shown as the average and variance of two measurements. The methods used in the kinase selectivity screen are described in Bain et al, Biochemical Journal, 2007, 408 (3); 297-315.
