# Supplementary material for: Dectin-1 Regulates IL-10 Production via a MSK1/2 and CREB Dependent Pathway and Promotes the Induction of Regulatory Macrophage Markers
Source: PLoS One. 2013 Mar 22;8(3):e60086. doi: 10.1371/journal.pone.0060086 (PMC3606242; doi:10.1371/journal.pone.0060086)
Supplement: Figure S2 — Effect of MAPK inhibitors and MSK1/2 knockout on TNF mRNA Induction. (A) Wild type BMDMs were treated with 2 µM PD184352 or 5 µM SB203580 as indicated. Cells were then stimulated for a further 1 h with 200 µg/ml zymosan. Total RNA was isolated and the levels of TNF mRNA determined by qPCR. (B) as (A) except cells were stimulated for 8 h with zymosan. (C) Wild type BMDMs were incubated for 1 h with 5 µM SB-747651A before stimulation with 200 µg/ml zymosan for a further 1 h. TNF mRNA levels were determined by qPCR. (D–F) Wild type or MSK1/2 knockout BMDMs were stimulated for between 1 and 12 h as indicated with either 200 µg/ml zymosan (D), 10 µg/ml curdlan (E) or 200 µg/ml depleted zymosan (F). Error bars represent the standard deviation of independent cultures from 4 mice per genotype. (PDF) [file pone.0060086.s002.pdf]

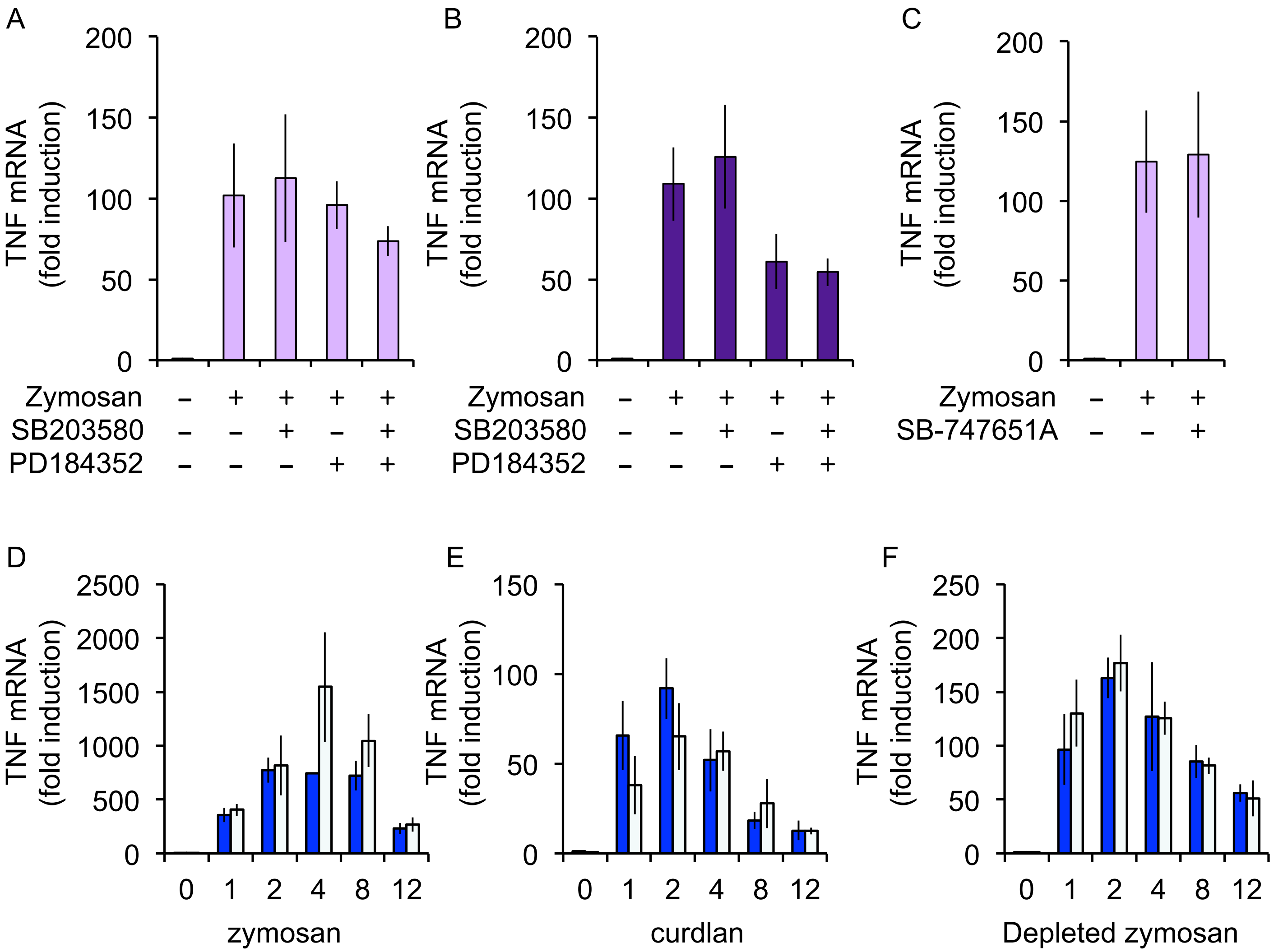

Figure S2. Effect of MAPK inhibitors and MSK1/2 knockout on TNF mRNA Induction.
(A) Wild type BMDMs were treated with 2 μM PD184352 or 5 μM SB203580 as indicated. Cells were then stimulated for a further 1 h with 200 μg/ml zymosan. Total RNA was isolated and the levels of TNF mRNA determined by qPCR. (B) as (A) except cells were stimulated for 8h with zymosan. (C) Wild type BMDMs were incubated for 1h with 5 μM SB-747651A before stimulation with 200 μg/ml zymosan for a further 1 h. TNF mRNA levels were determined by qPCR. (D-F) Wild type or MSK1/2 knockout BMDMs were stimulated for between 1 and 12 h as indicated with either 200 μg/ml zymosan (D), 10 μg/ml curdlan (E) or 200 μg/ml depleted zymosan (F). Error bars represent the standard deviation of independent cultures from 4 mice per genotype.
